# Arterial Hypertension in Aortic Valve Stenosis: A Critical Update

**DOI:** 10.3390/jcm10235553

**Published:** 2021-11-26

**Authors:** Christian Basile, Ilaria Fucile, Maria Lembo, Maria Virginia Manzi, Federica Ilardi, Anna Franzone, Costantino Mancusi

**Affiliations:** Department of Advanced Biomedical Science, Federico II University of Naples, 80131 Naples, Italy; christian.basile@unina.it (C.B.); fucile.ilaria@gmail.com (I.F.); maria.lembo@unina.it (M.L.); mariavirginia.manzi@unina.it (M.V.M.); fedeilardi@gmail.com (F.I.); anna.franzone@unina.it (A.F.)

**Keywords:** left ventricular remodeling, high blood pressure, antihypertensive drug, echocardiography

## Abstract

Aortic stenosis (AS) is a very common valve disease and is associated with high mortality once it becomes symptomatic. Arterial hypertension (HT) has a high prevalence among patients with AS leading to worse left ventricle remodeling and faster degeneration of the valve. HT also interferes with the assessment of the severity of AS, leading to an underestimation of the real degree of stenosis. Treatment of HT in AS has not historically been pursued due to the fear of excess reduction in afterload without a possibility of increasing stroke volume due to the fixed aortic valve, but most recent evidence shows that several drugs are safe and effective in reducing BP in patients with HT and AS. RAAS inhibitors and beta-blockers provide benefit in selected populations based on their profile of pharmacokinetics and pharmacodynamics. Different drugs, on the other hand, have proved to be unsafe, such as calcium channel blockers, or simply not easy enough to handle to be recommended in clinical practice, such as PDE5i, MRA or sodium nitroprusside. The present review highlights all available studies on HT and AS to guide antihypertensive treatment.

## 1. Introduction

Aortic stenosis (AS) is the most common heart valve disease in Western countries, with a prevalence that is increasing in tandem with life expectancy [1]. Arterial hypertension (HT), which becomes increasingly present as the population ages, is common among patients with AS [2].

HT interferes with both progression of AS and with the assessment of the severity of AS, leading to an underestimation of the real degree of stenosis [3,4].

The latest guideline on valvular heart diseases [1] reports safety of ACE-Is, but based on the available evidence, both angiotensin receptor-blocking agents and ACE-Is are well-studied and might be considered safe, while use of beta-blockers might be considered when indicated by compelling indications. Use of ambulatory blood pressure monitoring should be suggested based on the evidence that accurate BP control has a pivotal role in these patients [5]. The purpose of this document is to review the latest available evidence on the treatment of HT in patients with AS.

## 2. Epidemiology

AS is a very common valve disease, affecting between 2% and 4% of adults over the age of 65, and is associated with high mortality once it becomes symptomatic [1]. While tricuspid aortic valve degeneration is the most prevalent cause of AS in older individuals, bicuspid aortic valve degeneration is the most common cause of AS in younger people, with AS being one of the key determinants of ascending aortic dilatation in those patients [6,7]. HT has a high prevalence among patients with AS, leading to worse LV remodeling and faster degeneration and calcification of the valve [3]. Because the frequency of both HT and AS rise with age, the two disorders frequently coexist. A history of HT raises by 23% the relative risk of AS [8]. Long-term exposure to raised BP was related with an increased risk of AS in a cohort study of a population without previous known cardiovascular illness [9]; HT was also shown to be a prevalent comorbidity in up to 78% of older individuals with AS [10,11].

## 3. Pathophysiology

Several studies have found that a history of HT increases the risk of developing senile aortic sclerosis [12]. Cuniberti et al. [13] discovered that HT alone might cause disfunctions in the valve’s function and morphology. This could be driven by the fact that HT-induced hemodynamic flow disturbance can cause mechanical damage to the valve. The pathophysiology of AS is comparable to that of HT, and that could be the link between the two diseases; for example, both contain a strong activation of profibrotic and proinflammatory markers. The pathogenesis of AS includes increasing fibrosis and calcification as well as gradual decreases in valve area. Although it is usually assumed to be a degenerative condition, a progressive inflammatory process may also be involved. The amplification of fibrotic and inflammatory processes has an adverse effect on aortic valve remodeling and calcification. Increased oxidative stress and cytokines cause cell apoptosis, endothelial dysfunction and extracellular matrix formation. Aside from the involvement of the valve’s fibroblasts in the development of both AS and HT, the smooth muscle cells and the myofibroblasts in the valve might play a significant role since they control the valve’s tone, but their role is still not totally understood. Valvular endothelial dysfunction is thought to enhance lymphocyte and macrophage infiltration, which activate various profibrotic and proinflammatory cytokines and may regulate aortic valve remodeling and eventual calcification. Transforming growth factor β1 (TGF- β1) and interleukin-1β have been found in valve matrix [14,15] and are associated with increased local production of matrix metalloproteinases I and II. All of these cause cell apoptosis, extracellular matrix development and, eventually, valve calcification. Tenascin C, an extracellular matrix glycoprotein involved in cell proliferation, migration, differentiation and apoptosis, has also been linked to AS calcification and progression [16]. A vast body of evidence supports the notion that enhanced localized tenascin expression by vascular smooth muscle cells is related with HT and may facilitate angiotensin II-induced alterations in vascular structure [17]. All the aforementioned cause a number of pro-oxidants and a rise in oxidative stress in both HT and AS, as evidenced by a number of studies [18]. Angiotensin II, on the other hand, is a key mediator in the pathophysiology of AS, similar to HT. ACE has been found in stenotic aortic valves but not in normal ones [19]. Additionally, human AS valves contain a higher amount of cathepsin G, a protease capable of generating Ang II, than normal valves [20]. Finally, NO generation, which is a physiological regulator of both vasomotor tone and platelet aggregation, is reduced in AS. In patients with AS, plasma concentrations of asymmetric dimethylarginine, an inhibitor of NO synthase and a biomarker and modulator of endothelial dysfunction, are higher than in patients without AS [21]. Furthermore, HT contributes to the concomitant development of aortic calcification by promoting vascular calcium buildup. Angiotensin II-induced fibrosis and hypertrophy appear to contribute to left ventricular remodeling in both situations and could contribute to the disease evolution since ACE has been found in stenotic but not in normal aortic valves [22].

## 4. Combined Effects of Aortic Stenosis and Hypertension

**Figure 1:**
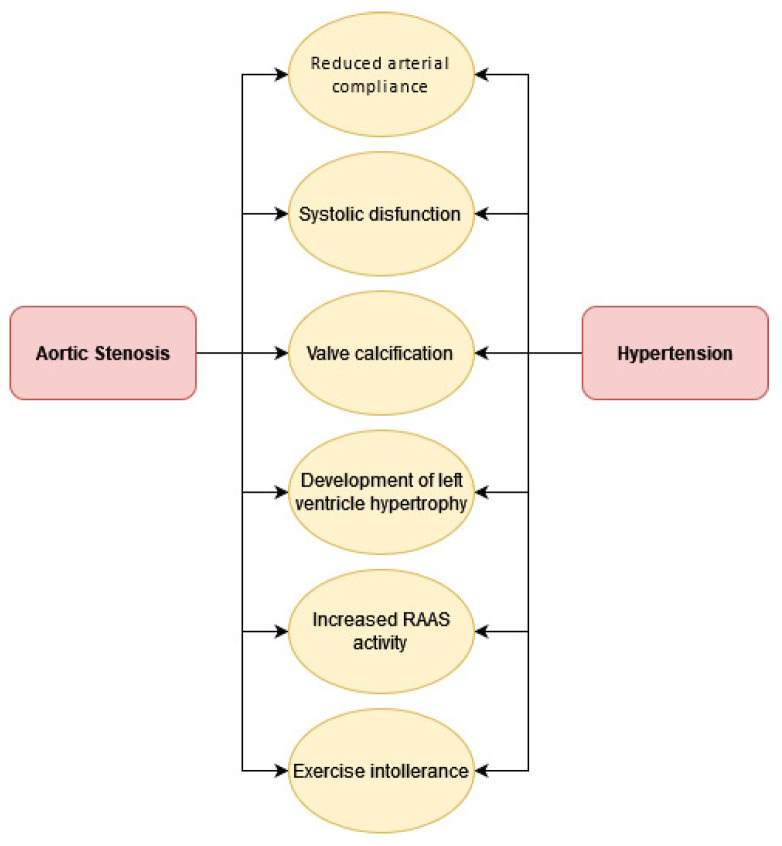
Connections between HT and AS.

Traditionally, the remodeling of the LV in AS has been considered as a compensatory response to the increased wall stress related to the severity of the valve obstruction. When HT coexists with AS, the LV is exposed to a greater hemodynamic load, so if the stenosis is moderate, ventricular remodeling is more influenced by comorbidities such as HT, and therefore, eccentric LV hypertrophy is the most frequent form of abnormal ventricular geometry [23,24,25,26]. In contrast, in patients with severe stenosis, remodeling is mainly driven by valve obstruction, and the most frequent geometric pattern is concentric hypertrophy [24]. In both cases, this subsequently leads to heart failure with severe diastolic dysfunction (Figure 1).

The combination of AS and HT is the association of a first, fixed mechanical obstruction of the aortic root and a second obstruction due to systemic vascular resistance. Consequently, a decrease in systemic vascular resistance through, for example, the administration of vasodilators, could theoretically cause a drop in systemic pressure due to the fixed mechanical obstruction given by the stenosis, which prevents an increase in cardiac output. This theory was the basis for avoiding vasodilators in patients with AS, but the most recent evidence challenges this model [1]. Studies that have evaluated the impact of antihypertensive treatment in patients with AS have shown efficacy and, above all, safety of therapy with the most common antihypertensive molecules, as will be explained later in Section 6.

As shown by Rieck, Åshild E. et al. [27], abnormal LV geometry can predict cardiovascular events in AS patients with HT or normotension. Combined HT was uniquely related with greater abnormal LV, likely suggesting the HT group’s increased global valvulo-arterial burden. The requirement for further coronary revascularization within or following aortic valve replacement was twofold higher, indicating that HT leads to atherosclerosis and subsequent coronary artery disease. Additionally, the HT group showed higher systemic arterial stiffness and significantly increased peripheral resistance indicating arteriosclerosis; nonetheless, HT did not forecast a higher risk of aortic valve replacement in the SEAS Study. These findings corroborate prior research on the topic, suggesting that AS should not be seen as a solitary valve disease but rather as an atherosclerotic disease involving the aortic valve as much as the systemic arteries [28,29].

Eleid et al. [30] investigated the relationship between HT and severe low-gradient (LG) AS in patients with preserved ejection fraction (EF), highlighting the relevance of evaluating arterial circulation features in addition to standard parameters in the evaluation of AS. HT was related with higher LV filling and pulmonary pressures in the context of severe LG AS with preserved EF, which were lowered by the vasodilator sodium nitroprusside.

Systolic dysfunction of the LV occurs before EF decreases in patients with AS. This dysfunction is highlighted both by LV global longitudinal strain (GLS) and by cardiac magnetic resonance studies [31,32]. Circumferential and longitudinal strains were also considerably reduced in AS patients in all three LV layers (endocardial, midmyocardial, epicardial) [33]. The mean gradient of the aortic valve and systolic blood pressure have been demonstrated to be independent of LV mass and EF but linked with LV GLS. This is critical because LV GLS has been linked to worse outcomes following aortic valve replacement (surgical or transcatheter) [34,35,36]. Significant variations in GLS were identified in normotensive and hypertensive patients with severe AS but not in those with moderate AS. Other variables, such as AS severity as defined by mean aortic valve gradient, may have a significant role in HT patients with severe AS having a more impaired longitudinal strain. However, the role of HT should not be overlooked. Because the hemodynamic measures of AS degree relies on flow, HT may have a major effect on the evaluation of AS severity by directly influencing the flow rate without affecting the aortic valve area. In the context of AS, HT increases afterload by raising systolic stress, lowering arterial compliance and increasing vascular resistance [37]. Measurement of blood pressure at time of echocardiography is of paramount importance since uncontrolled hypertension may lead to misdiagnosis [1].

These findings are linked to a significant dysfunction of the sympathetic nervous system, which in these regions presented the greatest mismatch between innervation and perfusion. This hypothesis is further supported by the nonreversibility of sympathetic dysfunction even after surgical treatment of the stenosis [38].

Other functional measures are severely impaired in patients with HT and AS [39]. Patients with HT reported increased resting systolic blood pressure (BP) before ETT, an accentuated BP response during ETT and delayed systolic BP regularization following ETT. All of these are known to be related with increased LV mass [40] and reflect a chronically raised BP overload on the LV and arterial system, necessitating prudent antihypertensive medication optimization to accomplish BP control. In contrast, disclosed symptoms during the ETT are independently related to a lower peak systolic BP and quick early increase in heart rate (HR), both of which are probably triggered by a decline in stroke volume during early exercise and a subsequent inability to rise [41]. A fast early elevation in HR has been linked to a poor prognosis in patients with moderate and severe AS [42]. The use of reported symptoms, alone, in asymptomatic patients with AS has been questioned due to the patient’s age, comorbidities and unreliability. ETT metrics such as age-adjusted metabolic equivalent of task (METs) and exercise length may deliver more objective and rigorous data on patient’s symptoms and functional status. Patients who did not exhibit symptoms disclosed by ETT had substantially higher peak systolic BP (inside the spectrum of physiological response), METs and lasted longer during ETT than those who did [40]. This demonstrates that patients with no symptoms have a higher exercise ability. Most notably, baseline HT, resting systolic BP prior to ETT and peak systolic BP during ETT had no effect on age-adjusted exercise length [42].

## 5. Challenges in Diagnosis

Attempts have been undertaken to evaluate total LV load more carefully in the context of AS and HT. Aside from the fixed obstruction a constricted valve, the systemic vascular resistance, which is determined by BP, arterial stiffness and vascular tone, is a critical factor on LV afterload. Valvulo-arterial impedance (Zva) evaluates the global LV hemodynamic load originating from the combination of the valvular and vascular loads [43] and reflects the variables that cause mechanical energy to be lost and converted into heat [44]. Zva is determined as the transvalvular pressure gradient (TVPG) + the systolic aortic BP/stroke volume index. Integrating the estimated stroke volume in this formula help compensate for transvalvular flow fluctuations [43,45]. Patients who have a greater TVPG or systolic pressure will have a higher Zva. Zva has been demonstrated to be independently linked with LV dysfunction as well as various other hemodynamic variables [43,46], which is most likely due to the combined effects of AS and HT in increasing the LV afterload. 

Carefully evaluating the degree of AS is critical for directing healthcare choices in patients with AS [47]. BP, like many other factors assessed by echocardiography or cardiac catheterization, can influence the evaluation and categorization of AS severity. Increased BP, which causes decreased arterial compliance, can greatly diminish the peak-to-peak gradient during heart catheterization in animal models of AS and HT caused by thoracic aortic binding [48].

Handgrip exercise or phenylephrine infusion were utilized to raise BP and systemic vascular resistance in an echocardiography-based study of AS patients. While the mean transvalvular flow rate decreased, the mean pressure gradient remained constant. There was an inversely proportional correlation between the variation in mean BP and the estimated aortic valve area [49]. This study reveals how acute BP rise and higher systemic vascular resistance might alter echocardiographic assessment of AS. These variations in BP are most likely due to changes in mean transvalvular flow rate, rather than an independent influence of systemic vascular resistance or arterial stiffness. As a result, depending on how these flow changes, the degree of stenosis may be over- or underestimated [50]. Another study of patients who had an echocardiography evaluation for symptomatic AS found that those with HT developed symptoms with bigger aortic valve areas than those without HT [51]. This implies that AS patients with HT are more likely to be diagnosed with “symptomatic AS”, which can have a substantial impact on their treatment strategy. 

## 6. Treatment

Treatment of HT in AS does not have clear guidelines available, but consensus documents have been developed to give guidance to clinicians [7]. 

There is, therefore, no clear therapeutic strategy, and data are based on the studies that previously had evaluated the treatment of HT in AS (Table 1). 

There is a consensus on maintaining BP values of 130-139 mmHg for systolic and 80-90 mmHg for diastolic BP, as they are associated with lower mortality [52], while there is not the same agreement on which drugs to adopt to achieve the aforementioned values in patients with combined HT and AS [1].

Renin–Angiotensin–Aldosterone system (RAAS) inhibitors are certainly the drugs of choice. Their cardioprotective, plaque stabilizing and antiarrhythmic effects make them the drug to be adopted in the first instance [53]. Furthermore, a recent meta-analysis associated RAAS inhibitors with improved clinical outcomes in patients with moderate or severe AS [54], and they have been proved to be well-tolerated [55].

RAAS inhibitors have also been associated with increased survival rates and greater LV mass reduction after surgical [56,57] and transcatheter [58,59,60] aortic valve replacement for severe AS.

Many of the studies that compared angiotensin-converting-enzyme inhibitors (ACE-Is) and angiotensin-II receptor antagonists (ARBs) gave conflicting results. This may depend on the different composition of the populations under examination, as well as the different observation periods and the different endpoints taken into consideration.

ACE-Is have a wider literature, while ARBs have additional properties for the purposes not only of pressure control but also of remodeling and long-term prognosis of patients with AS: the chymase, an enzyme present on stenotic aortic valves and able to synthesize angiotensin 2, is blocked by ARBs but not by ACE-Is [61].

Furthermore, ARBs have been associated with a significant reduction in valve remodeling and valve calcium [62,63,64]. This effect of the ARBs could be explained by their ability to block the RAAS pathway, which, at valve level, produces the blocking of the chymase, which prevents inflammation and consequent progressive valve fibro-calcification. ACE-Is and statins have been linked to better outcomes in patients with aortic sclerosis, including substantial decreases in admissions for coronary artery disease, hospitalizations for heart failure and for statins, alone, advancement to AS and overall mortality [65]. It is unknown if aortic sclerosis is a measure of higher mortality or has a direct impact on clinical outcome. Even so, it has been demonstrated that, once these patients are identified, therapy with statins or ACE-Is is linked with a decrease in cardiac events. The mechanisms behind the favorable benefits of statins and RAAS inhibitors reported in patients with aortic sclerosis are unclear; however, they may be dependent on the medications’ vascular and myocardial protective properties [66].

RAAS inhibitors have also been demonstrated to have a variety of positive qualities, including enhancing vasodilation, restricting neurohormonal activation, promoting endothelial function, slowing atherosclerosis development and reversing vascular remodeling [67,68,69]. Other studies have found ACE and angiotensin (AT) receptors’ upregulation in aortic valve lesions [19,23].

ACE-Is also shows effects on AS progression, whereas other antihypertensive medications, including ARBs, do not [70]. This result is consistent with a prior study that found a link between ACE-Is usage and a decreased rate of aortic valve calcium buildup [71].

Because both function similarly to inhibit the RAAS, one may anticipate ACE-Is and ARBs to affect AS development in a comparable manner. However, unlike ACE-Is, ARBs administration did not usually slow down the course of AS [72]. There are various likely possibilities for the distinction between ACE-Is and ARBs. Both ACE-Is and ARBs protect the cardiovascular system by blocking Ang II-induced activation of the angiotensin II type 1 (AT1) receptor by lowering Ang II production (ACE-Is) or by binding competitively to the AT1 receptor (ARBs). They do, however, have distinct properties in addition to inhibiting the Ang II–AT1 receptor pathway: ACE-Is promotes the bradykinin/nitric oxide pathway [73,74], inhibits matrix metalloproteinase (MMP) [75] and suppresses the AT2 receptor by decreasing Ang II, whereas ARBs activates AT2 receptor by raising plasma renin activity and Ang II [76].

All these elements combined make unclear the choice between the two classes of RAAS inhibitors.

If blood pressure is not yet controlled by RAAS blocking, the addition of a beta-blocker (BB) should be considered; among these, metoprolol has the greatest literature evidence, showing not only an improvement in hemodynamic and metabolic performance but also a reduction in mortality in patients who already presented with coronary artery disease [77,78].

BB therapy was also linked to decreased rates of cardiac and all-cause mortality, along with sudden cardiac death, and was not linked to an increased incidence of heart failure prior to AVR [79].

Despite a considerable drop in arterial blood pressure, there is no regression of LV mass in BB-treated patients with AS. There is evidence that severe valvular diseases cause humoral and cytokine activation comparable to heart failure, indicating that neurohormonal inhibition with BBs may play a role [80]. Finally, since major randomized controlled trials done in the 1970s and 1980s indicated significant decreases in mortality, largely due to sudden cardiac death, BB treatment has been a cornerstone in secondary preventive therapy for patients with CAD. As a result, even if SEAS patients did not have overt coronary disease, BB may have avoided clinical outcomes, owing to unrecognized concurrent coronary atherosclerosis.

Sodium nitroprusside, a predominantly arterial vasodilator, was evaluated in both normal flow AS and in patients with low-flow, low-gradient (LG) AS. Most patients in the study had a low stroke volume at baseline that increased after taking nitroprusside. This emphasizes the observation that patients with HT and severe LG AS with preserved EF have two obstacles in series and that treatment of HT may help lessen symptoms as well as cardiovascular risk. However, the available studies have been performed in hospitalized patients and under close clinical and laboratory monitoring of hemodynamic and blood pressure values; consequently, these drugs can be useful in case of exacerbations of heart failure in Coronary Care Units (CCU) or otherwise supervised settings [81,82].

Mineralocorticoid receptor antagonist (MRA) can be used. Among them, eplerenone has been studied. Its use has not been shown to slow the progression of AS, and too deliberate use can lead to a severe reduction in peripheral perfusion, given the state of dependence on preload of patients with AS. Thus, it can be useful in reducing the preload provided that close fluid and echocardiographic monitoring is implemented [83].

Several clinical studies have demonstrated that using phosphodiesterase 5 inhibitors (PDE5-i) is beneficial to the hemodynamic status of patients with AS and reduces LV hypertrophy, as well as improves pulmonary circulation and improves exercise tolerability of patients with AS [84,85,86]. In the study that specifically evaluated sildenafil in patients with AS, the safety and tolerability were evaluated in patients with severe AS, showing that, in the face of a reduction in the filling pressure, stroke volume increased despite the severe AS [87]. However, it should be considered that, in a subsequent study on the treatment of residual pulmonary hypertension in patients undergoing previous valve replacement treatment, the same sildenafil was associated with a worse clinical result compared to placebo [88]. The discrepancy between these two studies must also be taken into consideration when evaluating the different settings of use of the drug: in the first, sildenafil was administered in single doses under close clinical–instrumental monitoring to relieve symptoms; in the second study, it was given in chronic home therapy.

Calcium channel blockers (CCB) are one of the most regularly prescribed drugs for HT patients. According to Saeed et al. [89], the use of CCB in patients with moderate or severe asymptomatic AS was related with a sevenfold relative risk increase of all-cause death regardless of established confounders and prognosticators in AS. It was also linked to decreased treadmill activity and considerably greater risks of all-cause mortality regardless of age, HT and other covariates. 

The data on the subtypes of CCB (dihydropyridines versus non-dihydropyridines) and additional indications for its usage other than HT were not recorded, which was one of the study’s primary weaknesses. Moreover, the procedure for measuring BP prior to ETT was performed in accordance with clinical practice, in the presence of a nurse or exercise physiologist. This might be regarded as a restriction in terms of translation to the research setting. Finally, there were no data on cause-specific death. As a result, based on available evidence, CCB should be avoided.

Newer medications, on the other hand, may be beneficial in patients with both HT and AS. Angiotensin receptor neprilysin inhibitor (ARNi) is a new class of RAAS inhibitors that operates by concurrently inhibiting neprilysin and blocking the AT1 receptor via an ARB. The benefits of ARNi are related to the augmentation of peptides degraded by neprilysin, such as natriuretic peptides, and ARB’s simultaneous reduction of angiotensin II’s detrimental effects. They considerably reduced blood pressure more effectively in hypertensive patients than ARBs, alone, with no significant differences in side effects, while, when compared to ACE-I, they generated less bradykinin buildup and angioedema [90]. They also increased protection against heart functional decline in a pressure unloading animal model according to Li X et al. [91]. Cardiac fibrosis and inflammation were altered in debanding-surgery-treated mice hearts vs. aortic-binding-treated hearts, and these alterations were decreased further in the presence of ARNi medication. Furthermore, ARNi protected mice from myocardial fibrosis and inflammation after debanding surgery by inhibiting NF-κB-mediated NLRP3 inflammasome expression. Desai AS et al. [92] showed that, in patients with heart failure and reduced ejection fraction, treatment with ARNi did not significantly reduce central aortic stiffness compared with ACE-I. The paucity of literature regarding the use of ARNi in this context further highlights the need for further studies.

Sodium–glucose cotransporter 2 inhibitors (SGLT-2i) have been shown to minimize cardiovascular events, notably in heart failure. There is no obvious explanation for the cardioprotective actions of this novel family of medicines, but several hypotheses have been advanced. This impact might be attributed to the improvement of ventricular loading conditions through a reduction in preload caused by osmotic natriuresis induced by blocking SGLT2 reabsorption of glucose and afterload caused by a BP reduction and improved vascular function; the improvement of cardiac metabolism; or changes in cytokine production and epicardial adipose tissue mass. They could potentially be of great help in this setting due to their anti-remodeling effect, but there are currently no studies that have considered this class of drugs in patients with combined AS and HT. On the other hand, a new trial will evaluate the effects of SGLT-2i in patients with AS undergoing TAVI [93].

## 7. Conclusions

Target blood pressure in patients with concomitant HT and AS should be SPB 130–139 mmHg, and DBP, 80–90 mmHg. Treatment should begin with a low dose, followed by a planned titration that ensures regular patient assessment with the goal of applying tailored pharmacological choices to avoid hypotension.

RAAS inhibitors are well-tolerated and improve the clinical outcome both before and after valve replacement; beta-blockers are well-tolerated and may be considered, especially in patients with concomitant coronary artery disease, heart failure or arrhythmias.

**Figure 2:**
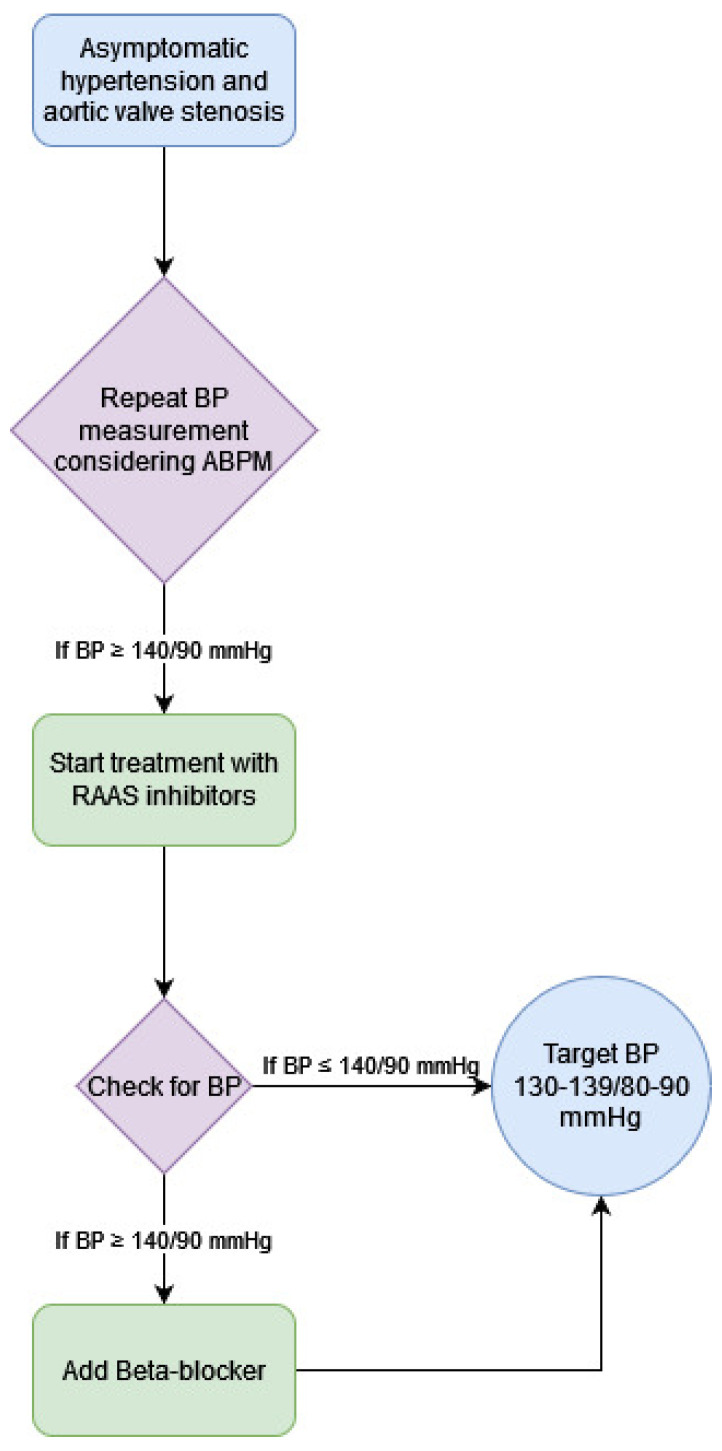
Proposed therapy scheme.

At present, the therapy we would like to recommend is displayed in Figure 2. 

Before being able to draw definitive conclusions on HT therapy in patients with AS, new studies and more evidence are needed.

## Figures and Tables

**Table 1:** Selected studies on the treatment of hypertension in aortic stenosis.

Trial, Author Year	Design	Sample Size	Medication or Class	Follow-Up	Results
Khot, U. et al., 2003 [82]	prospective	25	Nitroprussiate	24 h	Nitroprusside improves heart function in patients with decompensated heart failure due to severe left ventricular systolic dysfunction and severe aortic stenosis.
SCOPE-AS, Chockalingam et al., 2004 [55]	randomized double-blind	52	Enalapril 2.5 mg bis in die titrated up to 10 mg bis in die vs. placebo	12 weeks	NYHA class, Borg index and 6 min walking test improvement.
O’Brien et al., 2005 [71]	retrospective	123	ACE-inhibitors	2.6 ± 1.8 years	Less calcification of the aorta on CT.
Ralph A H Stewart et al., 2008 [83]	randomized	65	Eplerenone 100 mg/die	19 months	In patients with moderate–severe aortic stenosis, eplerenone does not slow down the onset of ventricular dysfunction, does not reduce the mass of the left ventricle and does not reduce the progression to valve stenosis.
Nadir et al., 2011 [53]	retrospective	2117	RAAS blockers	4.2 years	Lower frequency of mortality and cardiovascular events.
Lindman BR et al., 2012 [87]	open-label	22	Sildenafil 40 mg or 80 mg		A single dose of Sildenafil is safe and well-tolerated in patients with symptomatic severe aortic stenosis. It also improves stroke volume and reduces pre- and postload.
Eleid MF et al., 2013 [30]	prospective	24	Nitroprussiate		Nitroprusside is safe in patients with low-flow LG AS.
Capoulade et al., 2013 [63]	retrospective	338	RAAS blockers	6.2 ± 2.4 years	Angiotensin II receptor blocker I, but not ACE-I, was associated with slower progression of AS and lower mortality.
Dalsgaard et al., 2014 [67]	randomized	44	Trandolapril up to 2 mg/die	3 days	Blood pressure, peripheral resistance and left ventricular end-systolic volume were significantly reduced.
Goel et al., 2014 [56]	retrospective	1752	RAAS blockers	5.8 years	Better long-term survival after aortic valve replacement.
Bang et al., 2014 [68]	prospective	1873	RAAS blockers	4.3 ± 0.9 years	Slowed progression of the ventricular mass.
RIAS, 2015 [69]	randomized double-blind	100	Ramipril 10 mg vs. placebo	1 year	Improved systolic function, decreased left ventricular mass and slight reduction in left ventricular mass with Ramipril.
Helske-Suishko et al., 2015 [70]	randomized	51	Candesartan	5 months	No improvement.
Yamamoto et al., 2015 [64]	prospective	359	No intervention	3 years	Angiotensin II receptor blockers were associated with a smaller decrease in the indexed valve area in patients with AS jet velocity <2 m/s.
Claveau et al., 2015 [81]	retrospective	195	Nitrates		When nitroglycerin was used for acute pulmonary edema in patients with moderate and severe aortic stenosis, the risk of clinically detected hypotension as an adverse event was comparable to patients without aortic stenosis.
Bang et al., 2017 [79]	prospective	1873	Beta-blockers	4.3 ± 0.9 years	Lower mortality.
Magne et al., 2018 [57]	retrospective	508	RAAS blockers	4.8 ± 2.7 years	Better long-term survival after valve replacement.
Inohara et al., 2018 [58]	retrospective	21312	RAAS blockers	1 year	Lower mortality and lower risk of rehospitalization 1 year after TAVI.
Ochiai et al., 2018 [59]	retrospective	1215	RAAS blockers	1.1 years	Lower mortality and greater reduction in ventricular mass 1 year after TAVI.
SIOVAC 2018 [88]	randomized	200	Sildenafil	6 months	Worst clinical outcome of patients treated with Sildenafil compared to placebo.
Rodriguez-Gabella et al., 2019 [60]	retrospective	2785	RAAS blockers	3 years	Reduced cardiovascular mortality at 1 and 3 years after TAVI.
Saeed et al., 2020 [89]	retrospective	314	Calcium channel blocker	2.9 ± 2.9 years	Sevenfold increased risk of all-cause mortality.

## Data Availability

No new data were created or analyzed in this study. Data sharing is not applicable to this article.
